# The role of medial prefrontal cortex in early social cognition

**DOI:** 10.3389/fnhum.2013.00340

**Published:** 2013-07-05

**Authors:** Tobias Grossmann

**Affiliations:** Early Social Development Group, Max Planck Institute for Human Cognitive and Brain SciencesLeipzig, Germany

**Keywords:** infancy, development, prefrontal cortex, social cognition, fNIRS

## Abstract

One major function of our brain is to enable us to behave with respect to socially relevant information. Much research on how the adult human brain processes the social world has shown that there is a network of specific brain areas, also called the social brain, preferentially involved during social cognition. Among the specific brain areas involved in the adult social brain, functional activity in prefrontal cortex (PFC), particularly the medial prefrontal cortex (mPFC), is of special importance for human social cognition and behavior. However, from a developmental perspective, it has long been thought that PFC is functionally silent during infancy (first year of life), and until recently, little was known about the role of PFC in the early development of social cognition. I shall present an emerging body of recent neuroimaging studies with infants that provide evidence that mPFC exhibits functional activation much earlier than previously thought, suggesting that the mPFC is involved in social information processing from early in life. This review will highlight work examining infant mPFC function across a range of social contexts. The reviewed findings will illustrate that the human brain is fundamentally adapted to develop within a social context.

## Introduction

Humans possess a number of higher cognitive skills vital for language, reasoning, planning, and complex social behavior. The prefrontal cortex (PFC) can be seen as the neural substrate that underpins much of this higher cognition (Wood and Grafman, 2003). PFC refers to the regions of the cerebral cortex that are anterior to premotor cortex and the supplementary motor area (Zelazo and Müller, 2002). Based on its neuroanatomical connections, the PFC can be broadly divided into two sections: (a) the medial PFC (mPFC) and (b) the lateral PFC (lPFC) (Wood and Grafman, 2003; Fuster, 2008). The mPFC includes the medial portions of Brodmann areas (BA) 9–12, and BA 25, and has reciprocal connections with brain regions that are implicated in emotional processing (amygdala), memory (hippocampus) and higher-order sensory regions (within temporal cortex) (for more detailed information see, Wood and Grafman, 2003; Fuster, 2008). The lPFC includes the lateral portions of Brodmann areas (BA) 9–12, BA 44, 45 and BA 46, and has reciprocal connections with brain regions that are implicated in motor control (basal ganglia, premotor cortex, supplementary motor area), performance monitoring (cingulate cortex) and higher-order sensory processing (within temporal and parietal cortex) (for more detailed information see, Wood and Grafman, 2003; Fuster, 2008).

Critically, the distinction between lPFC and mPFC in neuroanatomical terms maps onto general differences in brain function. Namely, while mPFC is thought to be mainly involved in processing, representing and integrating social and affective information, lPFC is thought to support cognitive control process (Wood and Grafman, 2003; Fuster, 2008). This general functional distinction between mPFC and lPFC can already be seen early in development during infancy (Grossmann, 2013), thus representing a developmentally continuous organization principle of PFC function. As far as brain function is concerned, mPFC has been shown to play a fundamental role in a wide range of social cognitive abilities such as self-reflection, person perception, and theory of mind/mentalizing (Amodio and Frith, 2006). This involvement of mPFC in social cognition and interaction has lead to the notion that mPFC serves as a key region in understanding self and others (Frith and Frith, 2006). Although this is not the focus of this review, it should be noted that apart from its implication in social cognitive functions in adults, mPFC has been shown to be more generally involved in a number of processes related to decision making in adults (e.g., Heekeren et al., 2008). In particular, most recently, a unifying model has been proposed that views mPFC as a region concerned with learning and predicting the likely outcomes of actions (Alexander and Brown, 2011).

Only very little is known concerning the role of the mPFC in the development of social cognition. This is particularly true for the earliest steps of postnatal development, namely during infancy (the first year of life). Addressing the question of whether mPFC plays a role in infant social cognition and if it does, to theorize about what role this might be is the goal of this review. Such a look at early social cognition during infancy through the lenses of social neuroscience is critical because it allows us (a) to understand the nature and developmental origins of mPFC function, and (b) to close a gap between the extensive behavioral work showing rather sophisticated infant social cognitive skills (Spelke and Kinzler, 2007; Woodward, 2009; Baillargeon et al., 2010) and the social neuroscience work with adults studying mature mPFC functioning (Amodio and Frith, 2006; Lieberman, 2006).

That mPFC plays an important role in the development of social cognition is evident in work examining mPFC lesions. For example, there is work comparing early onset (during infancy) and adult onset lesions to mPFC (Anderson et al., 1999). This work shows that, despite typical basic cognitive abilities, patients with mPFC lesions had severely impaired social behavior. More specifically, regardless of when the mPFC lesion had occurred, there are symptoms shared across patients with mPFC damage, including an insensitivity to future consequences of actions, defective autonomic responses to punishment contingencies, and failure to respond to interventions that would change behavior (Anderson et al., 1999). Critically, this study revealed that over and above the shared symptomatology, acquired damage to mPFC during infancy had a much more severe impact on social functioning signified by striking defects concerning social and moral reasoning, leading to a syndrome that closely resembled psychopathy. In this study, it was found that early onset damage to mPFC was related to antisocial behaviors such as stealing, violence against persons and property, severe impairment of social-moral reasoning and verbal generation of responses to social situations. Specifically, in adults with early onset lesions to mPFC, moral reasoning was conducted at a much lower level than expected by their age, such that moral dilemmas were mainly approached from an egocentric perspective characterized by avoiding punishment. Furthermore, early onset damage of mPFC was related to a limited consideration of the emotional implications of one owns behavior for others and much fewer responses generated to resolve interpersonal conflict. This suggests that mPFC plays a critical role in the acquisition of social and moral behaviors already early during ontogeny. It further suggests that in contrast to many other brain regions where damage and especially damage early in ontogeny can be compensated (Thomas and Johnson, 2008), mPFC appears to be less plastic or more vulnerable. This in turn indicates that there might be a sensitive period in development during which mPFC is required to develop and learn socially and morally appropriate behaviors. Even though the study of patients with lesions to the mPFC is of great importance in illuminating mPFC function, patients with circumscribed mPFC lesions acquired during infancy, as reported by Anderson and colleagues (1999), are extremely rare and can hence only provide limited insights into these early stages of developing mPFC function. It is therefore all the more important to employ functional neuroimaging to shed light on the development of mPFC function during infancy if we wish to better understand its role in early social cognition.

Recent advances in applying functional imaging technology to infants, specifically, the advent of using functional near-infrared spectroscopy (fNIRS) has made it possible to study the infant brain at work. fNIRS is an optical imaging method that measures hemodynamic responses from cortical regions, permitting for the localization of brain activation (Lloyd-Fox et al., 2010). Other neuroimaging techniques that are well established in adults are limited in their use with infants because of methodological concerns. For example, functional magnetic resonance imaging (fMRI) requires the participant to remain very still and exposes them to a noisy environment. Although fMRI has been used with infants, this work is restricted to the study of sleeping, sedated or very young infants. The method of fNIRS is better suited for infant research because it can accommodate a good degree of movement from the infants, enabling them to sit upright on their parent's lap and behave relatively freely while watching or listening to certain stimuli. In addition, unlike fMRI, fNIRS systems are portable. Finally, despite its inferior spatial resolution also in terms of obtaining responses from deeper (subcortical) brain structures, fNIRS, like fMRI, measures localized patterns of hemodynamic responses in cortical regions, thus allowing for a comparison of infant fNIRS data with adult fMRI data. In the last decade, there has been a surge of fNIRS studies with infants, including a number of studies that have looked at PFC activation during a wide range of experimental tasks (for review, see Grossmann, 2013). In the following sections, I shall review the available experimental evidence that implicate mPFC in infant social cognition. This review is aimed at providing an overview of the range of social contexts during which infants employ the mPFC. The review of the empirical work is organized according to the two main sensory modalities (audition and vision) in which social stimuli were presented to infants. Following the presentation of the experimental evidence, I will discuss a number of issues that arise from these studies. Finally, based on these findings, I will outline an account of what role mPFC plays in the early development of social cognition during infancy.

## Overview of studies reporting mPFC activation in infants

Newborns enter the world with a number of behavioral biases that allow them to preferentially attend and respond to certain stimuli such as faces and voices (Grossmann and Johnson, 2007), suggesting that infants enter the world endowed with biases that allow them to preferentially engage with the social world. However, while these biases found in newborns may be a vital foundation for the emergence for the development of social cognitive skills, we are only beginning to understand what role prefrontal brain regions play in these early attempts of the developing infant to respond to her environment and organize her perceptual experiences.

### Audition

The human voice, apart from having obvious functions in linguistic communication, also carries a wealth of socially relevant information such as age, gender, and emotional state (Belin et al., 2004). Newborns have been shown to show significantly increased responses in mPFC to their own mother's voice reading a story in infant-directed speech (IDS) compared to their mothers reading the same story in adult-directed speech (ADS) (Saito et al., 2007). This indicates that newborn infants discriminate between these two forms of speech and dedicate increased mPFC processing resources to IDS, which is of high socio-affective relevance to the infant. In another study, Saito et al. (2007) also showed that mPFC activation can be obtained in response to non-maternal emotional speech. This finding suggests that it is the emotional tone of voice that characterizes positive affect in speech that drives this effect on mPFC in newborns.

Older infants (4–13 months of age) were presented with IDS and ADS sentences spoken by their own mother or a female stranger and prefrontal and temporal cortex responses were measured using fNIRS (Naoi et al., 2012). This study showed that while infants' temporal cortex discriminated between IDS and ADS regardless of speaker, PFC (including mPFC in the left hemisphere) was engaged only when the mother spoke with IDS. Together with the data from newborns presented above, this suggests that mPFC responses undergo change during infancy and become more finely tuned to the primary caregiver's voice. Indeed, in agreement with behavioral work showing that at the age of 7–9 months infants show the strongest preferences for their primary caregivers, prefrontal responses change during infancy such that at 7–9 months of age infants' prefrontal brain activity is most sensitive to their mothers' IDS.

### Vision

Another important area of investigation is the work on the perception of visual social stimuli. The human face provides the infant with a wealth of socially relevant information such as age, gender and emotional state. From birth, human infants preferentially attend to faces (Johnson and Morton, 1991). For example, Tzourio-Mazoyer et al. (2002) presented 2-month-old infants with a face or a control stimulus, while measuring brain activity using positron emission tomography (PET) (note that although PET is not commonly used with infants due to the fact that it exposes infants to small amounts of radiation, the infants scanned in this study were tested in an intensive care unit as part of a clinical follow-up). In this study, when viewing faces infants not only activated regions in temporal cortex involved in distinguishing faces from other visual stimuli but also showed activation within the mPFC in the right hemisphere. This suggests that already at this young age infants recruit parts of the so-called extended face processing network that are considered to be crucial in assigning social and affective significance to faces (Haxby et al., 2000).

An important communicative signal conveyed by faces is eye gaze. The monitoring of eye gaze direction is essential for effective social learning and communication among humans (Csibra and Gergely, 2009), with eye contact being one of the most powerful modes of establishing a communicative link between humans (Kampe et al., 2003). In an fNIRS study, 4-month-old infants watched two kinds of dynamic scenarios in which a face either established eye contact or averted its gaze followed by a smile (Grossmann et al., 2008). The results revealed that, similar to what is known from adults (Kampe et al., 2003; Pelphrey et al., 2004), processing eye contact activates not only superior temporal cortex implicated in processing information from biological motion cues but also the mPFC important for social and affective communication. Moreover, in the same study, measuring electrical brain responses over PFC in another group of 4-month-old infants showed that only a smile that was preceded by eye contact evoked increased PFC responses in 4-month-old infants (Grossmann et al., 2008), supporting the notion that already in infancy mPFC plays a role in interpreting social and affective information directed at the self.

That smiling at an infant while making eye contact is a powerful cue triggering mPFC activation has also been demonstrated in another fNIRS study (Minagawa-Kawai et al., 2009), in which 9- to 12-month-old infants were presented with videos of either their own mother or a female stranger smiling at them or looking neutrally at them. Smiling at the infants evoked greater activity in mPFC regardless of the familiarity with the face, suggesting that mPFC is flexibly employed during positive social interactions. Nevertheless, mPFC activity was significantly greater in response to the own mother smiling when compared to the female stranger smiling, suggesting that infants' mPFC responses are particularly sensitive to affective cues from the primary caregiver. Interestingly, in this study it was shown that mothers exhibited a very similar mPFC response when looking at their own infants' smiling, thus pointing to a shared neural mechanism engaged during social interaction between caregivers and infants.

Eye gaze also plays an important role in coordinating attention during triadic interactions between self, other, and the environment. During a typical triadic interaction, a person may establish eye contact with another person and then direct that person's gaze to an object or event. In a recent study, fNIRS was used to localize infant prefrontal brain responses during triadic social interactions (Grossmann and Johnson, 2010). The results showed that by the age of 5 months, infants are sensitive to triadic interactions and, like adults, they recruit a specific prefrontal region localized in a dorsal part of the PFC (at the border between mPFC and lPFC) in the left hemisphere only when engaged in triadic interaction with another person but not during the conditions that controlled for certain aspects of the social interaction but were not triadic in nature (Schilbach et al., 2010). Very recently, it was shown that mPFC is not only involved when an adult guides infant attention to an object through gaze behavior, but it is also implicated in infants' detection of when a social partner followed their own gaze to an object, suggesting that infants flexibly use this brain region to coordinate attention with others regardless of whether the interaction is initiated by others or by themselves (Grossmann et al., under review).

The finding that specific parts of the mPFC play a role in triadic interactions receives more support from recent work examining the perception of human action. In this study (Lloyd-Fox et al., 2011), when 5-month-old were presented with actions (hand movements) while being addressed through eye contact and thereby creating a triadic interaction, they showed increased activation within the mPFC. The same regions of the mPFC were not active when human actions that were purely dyadic in nature such as mouth movement or eye gaze shifts.

### Audition and vision

In adults, initiating a social interaction by eye contact and calling a person's name results in overlapping activity in the mPFC (Kampe et al., 2003), suggesting that, regardless of modality, the intention to make contact is detected by the same brain region. In a recent fNIRS study (Grossmann et al., 2010), 5-month-old infants watched faces that either signaled eye contact or directed their gaze away from the infant, and they also listened to voices that addressed them with their own name or another name, in order to examine the neural basis of detecting social interactive signals across modalities. The results of this study revealed that infants recruit adjacent regions in the mPFC when they process eye contact and their own name. Moreover, 5-month-old infants that responded sensitively to eye contact in the one mPFC region were also more likely to respond sensitively to their own name in the adjacent mPFC region as revealed in a correlation analysis, suggesting that responding to communicative signals in these two regions is functionally related. These fNIRS results suggest that infants at the age of 5 months selectively process and flexibly attend to social interactive signals across modalities.

## Discussion

This review presented an overview of the experimental evidence on infants' mPFC involvement during the processing of auditory and visual social information. We have seen that infants employ mPFC during a wide range of contexts, including the perception of emotional and infant-directed speech cues in the auditory domain and the perception of faces and eye gaze cues in the visual domain. These findings support the central thesis that mPFC is important from early in ontogeny, playing a vital role in the emergence of social cognitive abilities during infancy. This notion stands in contrast to the idea that mPFC matures late and only plays a role later in ontogeny when a more explicit understanding of the social world is achieved (Singer, 2006; Blakemore, 2008).

On the basis of the evidence presented above, it could even be argued that mPFC is more important earlier in development than later in development because it is critically involved in the acquisition of social cognitive abilities from birth and becomes less important once social cognitive and interactive abilities have been robustly acquired. That this might indeed be the case is evident in the mPFC lesion work presented earlier where it was shown that early onset compared to adult onset lesions to mPFC resulted in more severe outcomes in terms of social and moral impairments (Anderson et al., 1999). More support for this view of mPFC playing a greater role earlier in development comes from neuroimaging work on social cognition with adolescents, which shows that while the engagement of posterior regions of cortex increases with age, mPFC involvement in social cognitive tasks decreases with age during adolescence (for a review, see Johnson et al., 2009). This can be seen as evidence for a reduction of the involvement of mPFC in social cognition during development, which also concurs with another line of work demonstrating that prefrontal regions play a greater role during the acquisition of a new perceptual skill (Gilbert and Sigman, 2007). Therefore, one implication of the work presented here is that mPFC plays a role in the acquisition of social cognitive skills from early in ontogeny. In general, this notion is in line with views that conceive of infants as competent social learners, entering the world readily prepared for social interaction and social thinking (Meltzoff, 2007; Spelke and Kinzler, 2007; Csibra and Gergely, 2009).

But what is the functional role that mPFC takes on in the early development of social cognition during infancy? I would like to put forward the proposal that mPFC involvement in infancy (and beyond) is likely to be important for the detection of self-relevant information. This proposal is based on (a) the observed pattern of mPFC involvement in the studies reviewed above, and (b) an extensive body of evidence from prior work with adults, implicating mPFC in assessing and representing information with reference to the self (for a review, see Amodio and Frith, 2006). This proposal can thus be seen as a developmental extension of prior accounts of adult mPFC function into infancy. More specifically, as shown above, mPFC is involved in infants' responding to social interactive cues, which index that information is relevant to the self such as during the listening to infant-directed speech or their own name, perceiving eye contact, or experiencing a triadic interaction. This increased sensitivity to self-relevant information might serve critical learning functions because it highlights potentially useful information that others present to the infant (Sperber and Wilson, 1995; Csibra and Gergely, 2009). In support of this view, it has been shown that infants' learning is influenced and improved when they are addressed by infant-directed speech and eye contact (Singh et al., 2004; Senju and Csibra, 2008; Yoon et al., 2008). The mPFC might thus be involved in learning from others by detecting the relevance of others' actions with reference to the self. Obviously, this sensitivity to self-relevant information in infancy does not imply that infants have an explicit (conceptual) understanding of the self (Rochat, 2003, 2011). However, one may argue that the sensitivity to self-relevant information serves as a powerful foundation for developing a sense of self because it provides infants with the opportunity to experience when the self is being addressed in an interaction. In fact, it has been argued that early social interactions during infancy and the experiences gained therein can be considered the cradle of self development (Reddy, 2003).

One intriguing implication of this proposal is that by measuring mPFC involvement in a given context, one might be able to examine the extent to which an infant perceives information as self-relevant. For example, on a trial- by-trial basis one could look at infants' mPFC response to eye contact and then see whether or not infants are more likely to show gaze following in response to an eye gaze shift of a social partner. The prediction based on the proposal presented above is that on trials during which infants show mPFC involvement when seeing eye contact they should be more likely to gaze follow. In behavioral work, it has already been shown that infants are more likely to gaze follow when they had previously been presented with eye contact or heard infant-directed speech (Senju and Csibra, 2008), however, it is unclear what the underlying neural processes are that correlate with this behavioral phenomenon. Moreover, this proposed approach might also be useful in assessing inter-individual differences in the perception of relevance to the self in response to identical stimuli. In such a scenario, we might be able to identify infants that tend to show little sensitivity to perceptual social signals indicating self-relevance but also infants that are overly sensitive to social information even if it is not directed at them. The potential existence of extreme biases in either direction in early development might have serious detrimental effects on social development in the long term. For example, a strongly reduced sensitivity to self-relevant information might be linked to neurodevelopmental disorder such as autism, where it has been shown that lacking behavioral sensitivity to self-relevant signals such as eye contact and name cues are some of the earliest detectable warning signs for the later development of autism (Zwaigenbaum et al., 2005; Elsabbagh and Johnson, 2007; Elsabbagh et al., 2012). The development of biomarkers such as brain-based measures to guide an early identification of developmental disorders is still in its infancy but has been shown to be of great promise, especially when relying on measures that assess infants' responses to eye contact (Elsabbagh et al., 2012).

Despite the progress that has been made in elucidating the role of mPFC in early development, in order to gain a better and more complete picture of mPFC function in infancy, it is vital to address a number of outstanding issues. First, more work is needed to precisely map and compare activation within the mPFC across social tasks during infancy. Specifically, as far as the infant fNIRS data presented in the review is concerned, no standardized anatomical mapping of the functional activation in PFC has been employed that would allow us to compare and integrate the information about mPFC across studies and tasks in a meta-analysis. This issue becomes particularly important when one considers the fact that in adults there appear to be considerable functional divisions within mPFC (Amodio and Frith, 2006; Bzdok et al., 2013). First strives have been made at standardizing the analysis of infant fNIRS data that promise to provide a better basis for carrying out such comparisons (Cristia et al., 2013). Nonetheless, a remaining issue is the limited depth resolution of fNIRS, as commonly used in infant studies, that obtains most of the signal from superficial cortical structures but is virtually blind to deeper cortical sources (Lloyd-Fox et al., 2010). Second, there is very little work comparing mPFC activation across ages during infancy because most work is focused on one particular age group. The few studies that have looked at various age groups during infancy revealed intriguing insights into how mPFC function changes and becomes more finely tuned to social signals from the caregiver (Naoi et al., 2012). A systematic examination of mPFC function across infancy will provide important information concerning the functional specialization of this brain region. Third, another important aspect to consider is that while we have observed activation of individual mPFC regions during infancy, we do not know whether the activity of the mPFC and other brain regions is coordinated into functional networks as seen in adults. There is work using resting-state fMRI with infants indicating that some of the functional connections between certain parts of mPFC and posterior cortical regions known in adults are not yet developed in infants (Fransson et al., 2007). Furthermore, resting-state studies testing infants across various ages show that this long-range integration of cortical activity emerges throughout the first few years of life (Gao et al., 2009; Homae et al., 2010; Fransson et al., 2011). The relevance that these changes in resting-state activity have for infants' brain function while actively involved in one of the experimental tasks reviewed here is unclear, and requires attention in future work.

Taken together, the findings from the studies presented here provide evidence that mPFC plays an important role in social cognition from very early in development. Based on the reviewed experimental data, I put forward the proposal that mPFC involvement in social information processing in infancy is related to the detection of self-relevant information. This look at early social cognition through the lenses of social neuroscience allowed us to better understand the nature and developmental origins of mPFC function by closing a gap between the extensive behavioral work showing sophisticated social cognitive skills in infants and work with adults concerning the pertinent role of mPFC played in social cognition. It is my hope, that this review will further stimulate work illuminating the neural basis of social cognition in infancy and foster the crosstalk between developmental psychologists and social neuroscientists.

### Conflict of interest statement

The author declares that the research was conducted in the absence of any commercial or financial relationships that could be construed as a potential conflict of interest.
